# Measuring the Prevalence of Problematic Respondent Behaviors among MTurk, Campus, and Community Participants

**DOI:** 10.1371/journal.pone.0157732

**Published:** 2016-06-28

**Authors:** Elizabeth A. Necka, Stephanie Cacioppo, Greg J. Norman, John T. Cacioppo

**Affiliations:** 1 University of Chicago, Chicago, Illinois, United States of America; 2 University of Chicago Pritzker School of Medicine, Chicago, Illinois, United States of America; Tilburg University, NETHERLANDS

## Abstract

The reliance on small samples and underpowered studies may undermine the replicability of scientific findings. Large sample sizes may be necessary to achieve adequate statistical power. Crowdsourcing sites such as Amazon’s Mechanical Turk (MTurk) have been regarded as an economical means for achieving larger samples. Because MTurk participants may engage in behaviors which adversely affect data quality, much recent research has focused on assessing the quality of data obtained from MTurk samples. However, participants from traditional campus- and community-based samples may also engage in behaviors which adversely affect the quality of the data that they provide. We compare an MTurk, campus, and community sample to measure how frequently participants report engaging in problematic respondent behaviors. We report evidence that suggests that participants from all samples engage in problematic respondent behaviors with comparable rates. Because statistical power is influenced by factors beyond sample size, including data integrity, methodological controls must be refined to better identify and diminish the frequency of participant engagement in problematic respondent behaviors.

## Introduction

Concerns have been raised in recent years about the replicability of published scientific studies and the accuracy of reported effect sizes, which are often distorted as a function of underpowered research designs [1–4]. The typical means of increasing statistical power is to increase sample size. Although increasing sample size was once seen as an impractical solution due to funding, logistic, and time constraints, crowdsourcing websites such as Amazon’s Mechanical Turk (MTurk) are increasingly making this solution a reality. Within a day, data from hundreds of MTurk participants can be collected inexpensively (MTurk participants are customarily paid less than minimum wage; [5–9]). Further, data collected on MTurk have been shown to be generally comparable to data collected in the laboratory and the community for many psychological tasks, including cognitive, social, and judgment and decision making tasks [10–13]. This has generally been taken as evidence that data from MTurk are of high quality, reflecting an assumption that laboratory-based data collection is a gold standard in scientific research.

However, traditional samples may also be contaminated by problematic respondent behaviors, and such behaviors may not pervade all laboratory samples (e.g., campus or community) equally. Factors such as participant crosstalk (participant foreknowledge of an experimental protocol based on conversation with a participant who previously completed the task) and demand characteristics continue to influence laboratory-based data integrity today, despite nearly half a century of research dedicated to developing safe-guards which mitigate these influences in the laboratory [14]. Similarly, non-naïveté is also a problem among MTurk participants. MTurk participants perform experiments frequently, are familiar with common experimental paradigms, and select into experiments [15]. Further, they engage in some behaviors which might influence the integrity of the data that they provide: a significant proportion complete the same study multiple times, provide misleading information, find information regarding successful task completion online, and provide privileged information regarding studies to other participants [15–17], even when explicitly asked to refrain from cheating [17]. Thus, it is probable that engagement in problematic respondent behaviors occurs with non-zero frequency in both more traditional samples and newer crowdsourced samples, with uncertain effects on data integrity.

To address these potential concerns with participant behavior during studies, a growing number of techniques have been developed that help researchers identify and mitigate the influence of problematic procedures or participants. Such techniques include instructional manipulation checks (which verify that a participant is paying attention; [18–19]), treatments which slow down survey presentation to encourage thoughtful responding [13,20], and procedures for screening for participants who have previously completed related studies [15]. Although these techniques may encourage participant attention, the extent to which they mitigate other potentially problematic behaviors such as searching for or providing privileged information about a study, answering falsely on survey measures, and conforming to demand characteristics (either intentionally or unintentionally) is not clear based on the current literature.

The focus of the present paper is to examine how frequently participants report engaging in potentially problematic responding behaviors and whether this frequency varies as a function of the population from which participants are drawn. We assume that many factors influence participants’ average behavior during psychology studies, including the safe-guards that researchers typically implement to control participants’ behavior and the effectiveness of such methods, which may vary as a function of the testing environment (e.g., laboratory or online). However, it is beyond the scope of the present paper to estimate which of these factors best explain participants’ engagement in problematic respondent behaviors. It is also beyond the scope of the current paper to estimate how engaging in such problematic respondent behaviors influences estimates of true effect sizes, although recent evidence suggests that at least some problematic behaviors which reduce the naïveté of subjects might reduce effect sizes (e.g., [21]). Here, we are interested only in estimating the extent to which participants from different samples report engaging in behaviors that have potentially problematic implications for data integrity.

To investigate this, we adapted the study design of John, Loewenstein, & Prelec (2012) [22] in which they asked researchers to report their (and their colleagues’) engagement in a set of questionable research practices. In the present studies, we compared how frequently participants from an MTurk sample, a campus sample, and a community sample reported engaging in potentially problematic respondent behaviors while completing studies. We examined whether MTurk participants engaged in potentially problematic respondent behaviors with greater frequency than participants from more traditional laboratory-based samples, and whether behavior among participants from more traditional samples is uniform across different laboratory-based sample types (e.g., campus, community).

We also examined whether engagement in such potentially problematic behaviors was related to factors which have previously been demonstrated to be associated with differential participant engagement. Of particular interest was the extent to which participants’ beliefs that survey measures represent meaningful psychological phenomena, participants’ frequency of completing studies, and participants’ reliance on study compensation for their primary source of income could predict engagement in potentially problematic respondent behaviors. Previous work has demonstrated that participants with more positive attitudes towards the experiment and experimenter are more likely to adhere to demand characteristics [23] and that engaging with the scientific goals of a study predicts task persistence [24], indicating that the extent to which participants feel as though their participation is important may be a factor which influences their behavior during studies. We hypothesized that participants who do not think that survey measures represent meaningful psychological phenomena might engage more frequently in potentially problematic respondent behaviors while completing studies. Furthermore, research indicates that more prolific participants are less distracted and more involved with research than less prolific participants [15]; thus, we hypothesized that participants who complete more studies would engage less frequently in potentially problematic respondent behaviors. Finally, though previous work has found no effect of compensation levels on data quality [25,26], whether using compensation from studies as one’s primary form of income affects engagement in potentially problematic respondent behaviors is as of yet unclear. Though some work suggests that India-based MTurk participants, who are twice as likely to use MTurk as their primary form of income as American-based MTurk participants [27], provide lower quality data than American-based participants [26], the impact of using compensation as one’s primary form of income among American-based participants, as used here, has yet to be explored. Thus, we explored the effect of using compensation from studies as one’s primary form of income on engagement in potentially problematic respondent behaviors.

To summarize, in the present study, we examined how frequently participants reported engaging in potentially problematic respondent behaviors and compared between an MTurk, campus, and community sample. Furthermore, we tested the extent to which a number of factors predict engagement in such behaviors.

## Methods

### Participants

#### MTurk Sample

MTurk participants (*N* = 870) who reported living in the US and being at least 18 years of age, and who had at least a 95% MTurk approval rating and had completed at least 1,000 approved studies on MTurk completed an online survey in Spring of 2014 regarding how MTurk participants perceive and respond to surveys from researchers in the behavioral sciences. These criteria were the default settings on MTurk at the time the study was run, are recommended in the Amazon Mechanical Turk Requester UI Guide [28], and are frequently used by researchers (see, for example, [29–30]). The study took an average of 7 minutes and 36 seconds to complete (*SD* = 296 seconds) and respondents were paid $0.75 (approximately $6/hour) for completing the study.

#### Campus Sample

Campus participants were recruited through flyers on the University of Chicago campus, ads on the University of Chicago’s online marketplace, and through posting of available timeslots on the University’s Psychology Department research participation system. Eighty-eight participants enrolled in the study before the end of the Spring of 2015 academic term, at which point data collection ceased. (Sample characteristics change dramatically in the summer, such that undergraduates comprise a substantially smaller portion of the campus recruitment pool). Participants were paid $3 or course credit for their participation (an approximate rate of $12/hour). Although this is nearly twice the rate that MTurk participants were paid, this payment discrepancy reflects the typical market rate for participation compensation for each of the samples and is common in similar designs which compare MTurk to other samples (e.g., [17]). Participants had to be at least 18 years of age and to have completed at least one laboratory study in the Psychology Department.

#### Community Sample

Community-based participants (*N* = 100) were recruited through email listings to the Booth Chicago Research Lab’s participant pool and posting of available timeslots on the Booth Chicago Research Lab’s research participation system in Spring of 2015. Participants from this community pool are members of the general Chicago public and are typically more diverse than a campus recruitment pool. As with the campus sample, participants were at least 18 years of age and had completed at least one study in the community testing environment. Participants were paid $3 for participation.

#### Sample size determinations and exclusion criteria

A priori sample size considerations were made to achieve adequate power, (1- *β*) = .80, to test an auxiliary hypothesis which is not presented in the current analyses. Data collection was originally limited to the MTurk sample, and we assumed a small effect (*d* = .20) and that 10% of participants would be excluded for poor data quality. The campus and community samples were originally conceived of as separate studies which would utilize the same procedure to test the hypothesis on a different population, and as such, sample size decisions were made to detect an effect the same size as the average effect size observed in the MTurk sample (*d* = .58). Thus, the desired sample size for the campus and community samples was 96 participants (48 participants per group).

Subjects were excluded if they met one of the following a priori exclusion criteria: a) incorrect answers to both of two instructional manipulation checks, b) an incorrect answer to one instructional manipulation check and evidence of straight-line responding, c) reported age less than 18 years old, and d) location outside of the US (for MTurk participants only. Location estimates were derived from IP addresses using the Qualtrics GeoIP feature). These exclusion criteria resulted in the exclusion of data from 22 MTurk participants (2.25%), no campus participants, and one community participant. However, four campus participants were excluded due to survey presentation error and one community participant was excluded on the basis of previously being included in the campus sample. Thus, analyses were conducted on 1,030 participants: 848 MTurk participants aged 18–81 years (*M* = 35.53, *SD* = 11.91, 407 males, 300 females; demographic information on some participants was not retained due to survey error), 84 campus participants aged 18–38 years (*M* = 21.27, *SD* = 3.50, 41 males, 43 females), and 98 community-based participants aged 19–82 years (*M* = 33.68, *SD* = 12.67, 57 males, 41 females). Full demographic characteristics of the samples are presented in Table 1.

**Table 1:** Demographic Comparison Between Samples.

	MTurk Sample		Campus Sample		Community Sample	
Demographics	*n*	*M (SD)*	*n*	*M (SD)*	*n*	*M (SD)*
Age		35.5 (11.9)		21.3 (3.5)		33.7 (12.7)
Gender						
Male	407		41		57	
Female	300		43		41	
Years of Education		15.1 (2.2)		14.2 (1.9)		15.6 (2.9)
Ethnicity						
African American	37		8		55	
American Indian/Alaskan Native	3		0		3	
Asian	50		25		4	
Caucasian	563		33		24	
Native Hawaiian/Pacific Islander	3		0		0	
Hispanic	34		10		7	
More than one race	14		7		1	
Other	3		1		4	
Marital Status						
Married	240		0		6	
Cohabitating	88		2		5	
Separated	4		1		2	
Divorced	50		0		10	
Widowed	5		1		1	
Never Married	320		80		74	

Survey presentation error led to lost demographic information on some participants in the MTurk sample.

### Procedure

All procedures were approved by the University of Chicago IRB. Participants read and signed an informed consent document that specified they would be compensated for their participation as long as they completed the study.

Participants then saw a list of problematic responding behaviors (see Table 1) and were randomly assigned to either report how frequently they engaged in each behavior (*frequency estimate for self* condition) or to report how frequently other participants engaged in each behavior (*frequency estimates for other* condition, similar to the manipulation used by [22]). We included a condition in which we asked participants to report on the behavior of other participants rather than themselves because we reasoned that participants may have been motivated to misreport their behavior (under-reporting engagement in socially undesirable respondent behaviors and over-reporting engagement in socially desirable respondent behaviors) if they inferred that their responses could influence future opportunities for paid participation in research (c.f. [31–32]). We expected that participants’ inferences of others’ behaviors would be egocentrically anchored upon their own behavior [33] but less influenced by self-serving reporting biases [34,35] and so could serve as more precise estimates of their own behavior.

In the *frequency estimate for self* (FS) condition (*N*_MTurk_ = 425, *N*_*Campus*_ = 42, *N*_Community_ = 49), participants reported how frequently they engaged in each problematic responding behavior. Specifically, participants were asked, “When completing behavioral sciences studies [*on MTurk / at the Psychology Department of the University of Chicago / at the Booth Chicago Research Lab*], what percentage of the time that you have spent [*on MTurk / completing studies*] have you engaged in each of the following practices?”

In the *frequency estimate for others* (FO) condition (*N*_MTurk_ = 423, *N*_*Campus*_ = 42, *N*_Community_ = 49), participants rated how frequently the average participant engaged in each problematic responding behavior. Specifically, participants were asked, “When completing behavioral sciences studies [*on MTurk / at the Psychology Department of the University of Chicago / at the Booth Chicago Research Lab*], what percentage of time spent [*on MTurk / completing studies*] does the average [*MTurk / research / Booth research*] participant spend engaging in each of the following practices?”

In the MTurk sample, which was collected before data collection from the campus and community samples began, we collected an additional 432 participants for a third condition in which participants rated how prevalent each problematic responding behavior was among other participants. We chose not to include this condition in the campus or community samples because it neither directly assessed participants’ own behavior nor could be used statistically to test the auxiliary hypothesis which is not presented in the current manuscript. In the campus and community samples, we also collected information about the frequency with which participants engaged in six additional behaviors, which were unrelated to completing psychology studies, to test the auxiliary hypothesis. Neither these questions nor the third MTurk condition are assessed further in the present manuscript.

Because we were interested in which factors might moderate participants’ engagement in each of the problematic responding behaviors, we also asked participants to answer a number of questions designed to assess their perceptions of psychological studies, frequency of completing studies, and financial incentives for completing studies. First, participants reported the extent to which survey measures represent a legitimate investigation of meaningful psychological phenomena. In the FS condition, participants reported what percent of the time that *they* believed that survey measures [*on MTurk / in psychology studies / in Booth research studies*] represented meaningful psychological phenomena. In the FO condition, participants reported what percent of the time that *the average* [*MTurk / Psychology Department / Booth research*] participant believed that survey measures [*on MTurk / in psychology studies / in Booth research studies*] represent meaningful psychological phenomena.

Next, participants in the FS condition reported whether or not they relied on [*MTurk / Psychology Department studies / Booth research studies*] as their primary form of income (yes or no) and how many hours a week they spent [*completing HITS on MTurk / completing studies in the Psychology Department/ completing studies at the Booth Chicago Research Lab*]. Participants in the FO condition instead reported what percentage of [*MTurk / Psychology Department research / Booth research*] participants relied on [*MTurk / compensation from Psychology Department studies / compensation from Booth research studies*] as their primary form of income, and reported how many hours a week the average [*MTurk / Psychology Department research / Booth research*] participant spent [*completing HITs on MTurk / completing studies in the Psychology Department / completing studies at the Booth Chicago Research Lab*].

All participants also reported whether or not each of the behaviors listed in Table 1 was defensible among MTurk, Psychology Department research, or Booth research participants (on a scale of No = 1, Possibly = 2, or Yes = 3), with the opportunity to explain their response in a free-response box. Because these data were intended to help test the auxiliary hypothesis which is not the focus of the present manuscript, these data are not presently analyzed further. Summaries of the qualitative data are available in the S1 File.

Finally, participants answered two items to assess their numeracy ability with percentages, as people with higher numeracy abilities tend to be more accurate in their frequency-based estimates [36]. Participants reported what percent 32 is of 100 and what percentage of time a standard American quarter would come up heads, using the same scale as they used in reporting how frequently they engaged in potentially problematic respondent behaviors. We reasoned that if participants successfully completed these problems, then there was a strong chance that they were capable of accurately responding to our percentage response scale as well. Throughout the study, participants completed three instructional manipulation checks, one of which was disregarded due to its ambiguity in assessing participants’ attention.

All items assessing percentages were assessed on a 10-point Likert scale (1 = 0–10% through 10 = 91–100%).

### Data reduction and analysis and power calculations

Responses on the 10-point Likert scale were converted to raw percentage point-estimates by converting each response into the lowest point within the range that it represented. For example, if a participant selected the response option 21–30%, their response was stored as the lowest point within that range, that is, 21%. Analyses are unaffected by this linear transformation and results remain the same if we instead score each range as the midpoint of the range. Point-estimates are useful for analyzing and discussing the data, but because such estimates are derived in the most conservative manner possible, they may underrepresent the true frequency or prevalence of each behavior by up to 10%, and they set the ceiling for all ratings at 91%. Although these measures indicate whether rates of engagement in problematic responding behaviors are non-zero, some imprecision in how they were derived limits their use as objective assessments of true rates of engagement in each behavior.

We combined data from all three samples to determine the extent to which engagement in potentially problematic responding behaviors varies by sample. In the laboratory and community samples, three items which were presented to the MTurk sample were excluded due to their irrelevance for assessing problematic behaviors in a physical testing environment. Further, approximately half of laboratory and community samples saw wording for two behaviors that was inconsistent with the wording presented to MTurk participants, and were excluded from analyses on these behaviors (see Table 1).

In all analyses, we controlled for participants’ numerical abilities by including a covariate which distinguished between participants who answered both numerical ability questions correctly and those who did not (7.3% in the FS condition and 9.5% in the FO condition). To compare samples, we conducted two separate analysis of variance analyses, one on the FS condition and another on the FO condition. We chose to conduct separate ANOVAs for each condition rather than a full factorial (i.e., condition x sample) ANOVA because we were primarily interested in how reported frequency of problematic responding behaviors varies by sample (a main effect of sample). It is possible that the samples did not uniformly take the same approach to estimating their responses in the FO condition, such significant effects of sample in the FO condition may not reflect significant differences between the samples in how frequently participants engage in behaviors. For example, participants from the MTurk sample may have considered that the ‘average’ MTurk participant likely exhibits more potentially problematic respondent behaviors than they do (the participants we recruited met qualification criteria which may mean that they behave better average, [37]) and responded accordingly, rather than anchoring on their own behavior and adjusting, whereas we expect participants from our campus and community samples would have anchored and adjusted because they are likely more similar to the ‘average’ participant in those samples. Thus, we chose to conduct separate models for the FS and the FO condition so as to isolate potential problems with the FO condition from contaminating results of the FS condition. Note that because we conducted separate models for each condition, any comparisons between the two conditions are not based on statistical comparison.

Comparisons between samples were made using two orthogonal contrasts, the first comparing the MTurk sample to the average of the campus and community samples to determine how crowdsourced samples differ from more traditional laboratory-based samples, and the second comparing the laboratory-based community and campus samples to determine if these behaviors are equally pervasive across different traditional samples. Because we were interested in generalizing our findings to research typically conducted in the social sciences, we compare MTurk participants’ behavior as they complete studies, by necessity, online, with campus and community participants’ behavior as they complete studies in traditional, physical laboratory testing environments. It is important to note, however, that this limits our ability to disentangle the influence of sample and mode of survey administration in our first orthogonal contrast.

Based on our final sample size, we had (1-β) = .80 power to detect a small to medium-sized effect (Cohen’s *d* = .33) in our between-sample comparisons in our first orthogonal contrast and (1- β) = .80 power to detect a medium-sized effect (Cohen’s *d* = .60) in our second orthogonal contrast. We also examined the extent to which the engagement in problematic respondent behaviors was associated with beliefs in the meaningfulness of survey responses in psychological investigations, time spent completing HITs or studies, or use of MTurk or research studies as primary income in each sample by conducting a multiple linear regression analysis on each problematic responding behavior. Statistical significance for all analyses was determined after controlling for a false discovery rate of 5% using the Benjamini-Hochberg procedure at the level of the entire paper.

## Results

Table 2 presents frequency estimates based on self-admission (FS condition) and assessments of other participants’ behavior (FO condition).

**Table 2:** Mean Frequency of Engagement in Potentially Problematic Responding Behaviors.

	MTurk Sample		Campus Sample		Community Sample	
Reporting Practice	Frequency		Frequency		Frequency	
	Other	Self	Other	Self	Other	Self
Begins studies without paying full attention to the instructions?	31.3% (24.2%)	10.2% (16.7%)	33.6% (20.4%)	13.0% (16.2%)	28.6% (28.8%)	12.2% (23.3%)
Responds without really thinking about a question?	26.8% (21.6%)	8.6% (14.1%)	35.5% (17.5%)	16.4% (14.5%)	27.6% (25.8%)	6.9% (17.8%)
Responds to questions in ways that are not entirely truthful?	24.0% (21.4%)	5.8% (13.4%)	26.1% (17.5%)	8.4% (9.4%)	25.3% (26.8%)	9.0% (23.6%)
Responds in ways that they deem to be socially acceptable? ^a^	45.2% (26.4%)	34.5% (36.4%)	50.6% (29.4%)	38.6% (34.8%)	46.6% (34.0%)	31.8% (39.2%)
Responds in a way that helps the researcher find support for his or her hypotheses? ^a^	46.3% (31.6%)	32.3% (37.5%)	29.0% (28.8%)	17.6% (30.2%)	41.6% (31.9%)	33.9% (38.8%)
Falsely reports the frequency with which they engage in certain behaviors?	21.6% (21.0%)	3.6% (10.4%)	24.9% (18.0%)	6.3% (12.4%)	20.0% (22.1%)	4.1% (11.1%)
Falsely reports one's age?	12.3% (17.1%)	2.2% (10.2%)	4.5% (12.2%)	0.3% (1.7%)	7.7% (15.0%)	1.9% (10.5%)
Falsely reports one's ethnicity?	10.2% (17.0%)	1.6% (9.0%)	4.3% (9.7%)	1.0% (6.3%)	7.0% (13.8%)	1.0% (7.3%)
Falsely reports one's gender?	8.9% (15.5%)	1.2% (6.6%)	0.8% (3.6%)	0.0% (0.0%)	4.2% (12.9%)	0.2% (1.6%)
Uses a search engine to find the answer to a survey or the key to an experimental task? ^b^	16.7% (20.7%)	4.5% 13.5%)	5.0% (11.4%)	0.0% (0.0%)	13.8% (24.2%)	3.6% (13.6%)
Spoken to other research participants to find answers to a survey or how to complete a task? ^c^	21.1% (26.3%)	5.8% (16.6%)	5.9% (9.8%)	0.5% (2.4%)	11.4% (19.9%)	3.0% (10.3%)
Provides privileged information (e.g. answers or instructions on how to complete a certain task) to other research participants? ^d^	11.7% (18.4%)	2.9% (12.2%)	9.8% (14.9%)	1.5% (6.7%)	18.4% (27.4%)	6.6% (21.1%)
Completes studies while multitasking (e.g. listening to music, checking one’s cell phone, etc.)? ^e^	41.2% (25.5%)	21.0% (24.0%)	11.3% (14.2%)	2.0% (7.0%)	27.8% (28.6%)	8.5% (19.0%)
Leaves the page of a study and returns at a later point in time?	27.1% (22.2%)	11.1% (15.3%)	12.0% (13.2%)	3.2% (12.0%)	13.5% (20.4%)	3.0% (10.3%)
Intentionally participates in the same study more than once?	11.1% (16.9%)	2.9% (9.9%)	7.5% (13.1%)	0.0% (0.0%)	7.0% (14.7%)	1.9% (8.0%)
Uses more than one [name when signing up for studies]? ^f^	4.9% (11.8%)	0.8% (6.7%)	4.3% (8.1%)	0.0% (0.0%)	9.8% (19.8%)	0.6% (4.4%)
Uses a VPN to appear to have a US IP address? ^g^	7.7% (13.4%)	0.9% (6.7%)				
Completes studies while completely alone? ^g^	61.0% (21.9%)	73.4% (23.6%)				
Completes studies while in the presence of others? ^g^	26.8% (21.1%)	15.8% (21.9%)				
Completes studies in a sleepy state?	26.3% (20.4%)	12.2% (15.8%)	41.0% (20.2%)	32.0% (25.1%)	21.9% (22.8%)	11.2% (17.7%)
Completes studies under the influence of alcohol or other drugs?	12.7% (16.7%)	3.8% (12.2%)	4.4% (7.1%)	2.4% (11.9%)	13.1% (20.2%)	3.5% (14.2%)
Looks for studies by a researcher that they already know?	56.2% (25.6%)	34.1% (29.1%)	17.4% (20.1%)	3.0% (8.1%)	10.9% (20.6%)	5.8% (17.4%)
Thoughtfully reads each question in a survey?	64.2% (19.4%)	80.2% (16.4%)	55.7% (20.2%)	71.2% (22.2%)	52.6% (27.8%)	76.8% (28.3%)
Contacts a researcher if there was a glitch with their survey?	50.5% (28.0%)	32.9% (33.5%)	51.1% (33.4%)	19.9% (33.1%)	44.2% (35.1%)	19.4% (32.5%)
Participates in a survey because the topic is interesting?	49.4% (25.6%)	43.5% (27.4%)	38.1% (23.5%)	43.7% (29.8%)	44.3% (32.3%)	43.2% (35.4%)

Standard deviations are listed in parentheses. All frequency estimates are percentages. In all materials for the MTurk sample, we called participants “workers” and researchers “requesters” in order to adhere to the terminology used by MTurk.

^a^ Approximately half of laboratory and community samples saw wording for these behaviors that was inconsistent with the wording presented to MTurk participants and were excluded from analyses on these behaviors.

^b^ For MTurk participants, we clarified that this excluded online forums such as TurkOpticon or TurkerNation

^c^ For MTurk participants, “spoken to other research participants” was replaced with “uses TurkOpticon, TurkerNation, or another forum”

^d^ For MTurk participants, “to other research participants” was replaced with “on forums such as TurkOpticon or TurkerNation”

^e^ For MTurk participants, “watching TV” was included as an example

^f^ For MTurk participants, this question stated “Uses more than one MTurk worker ID account.” For campus- and community-based participants, this stated “Uses more than one name when signing up on SONA”

^g^ For campus- and community-based participants, these items were excluded due to their irrelevance to assessing problematic responding behaviors in a physical testing environment

### Engagement in potentially problematic respondent behaviors across samples

#### FS Condition

**Fig 1:**
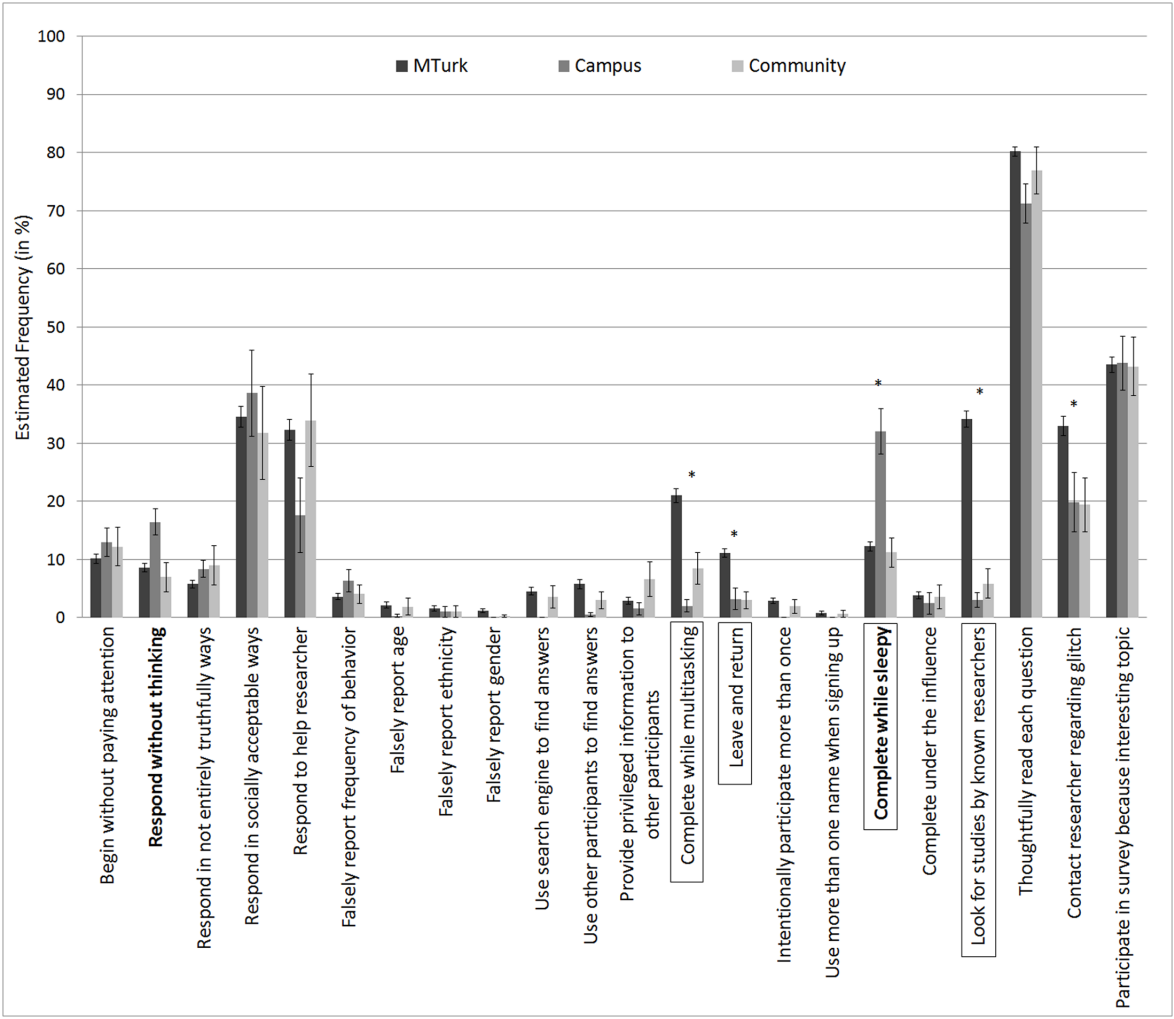
Estimates of the frequency of problematic respondent behaviors based on self-estimates. Error bars represent standard errors. Behaviors for which MTurk participants report greater engagement than more traditional samples are starred. Behaviors for which campus and community samples vary are bolded. Behaviors which vary consistently in both the FO and the FS condition are outlined in a box. Significance was determined after correction for false discovery rate using the Benjamini-Hochberg procedure. Note that frequency estimates are derived in the most conservative manner possible (scoring each range as the lowest point of its range), but analyses are unaffected by this data reduction technique. For complete text of each behavior, see Table 1.

We began by analyzing the effect of sample for participants in the FS condition (Fig 1). In the FS condition, significant differences emerged for the following potentially problematic respondent behaviors. The first orthogonal contrast revealed that MTurk participants were more likely than campus and community participants to complete a study while multitasking (*t*(512) = -5.90, *p* = 6.76E-9, *d* = .52), to leave the page of a study to return at a later point in time (*t*(512) = -4.72, *p* = 3.01E-6, *d* = .42), to look for studies by researchers they already know (*t*(512) = -9.57, *p* = 4.53E-20, *d* = .85), and to contact a researcher if they find a glitch in their survey (*t*(512) = -3.35, *p* = .001, *d* = .30). MTurk participants were less likely than campus and community participants to complete studies while sleepy (*t*(512) = 4.69, *p* = 3.51E-6, *d* = .41). The second orthogonal contrast revealed that campus participants were more likely than community participants to respond without thinking (*t*(512) = 3.26, *p* = .001, *d* = .29) and to complete studies in a sleepy state (*t*(512) = 5.73, *p* = 1.69E-8, *d* = .51).

#### FO Condition

**Fig 2:**
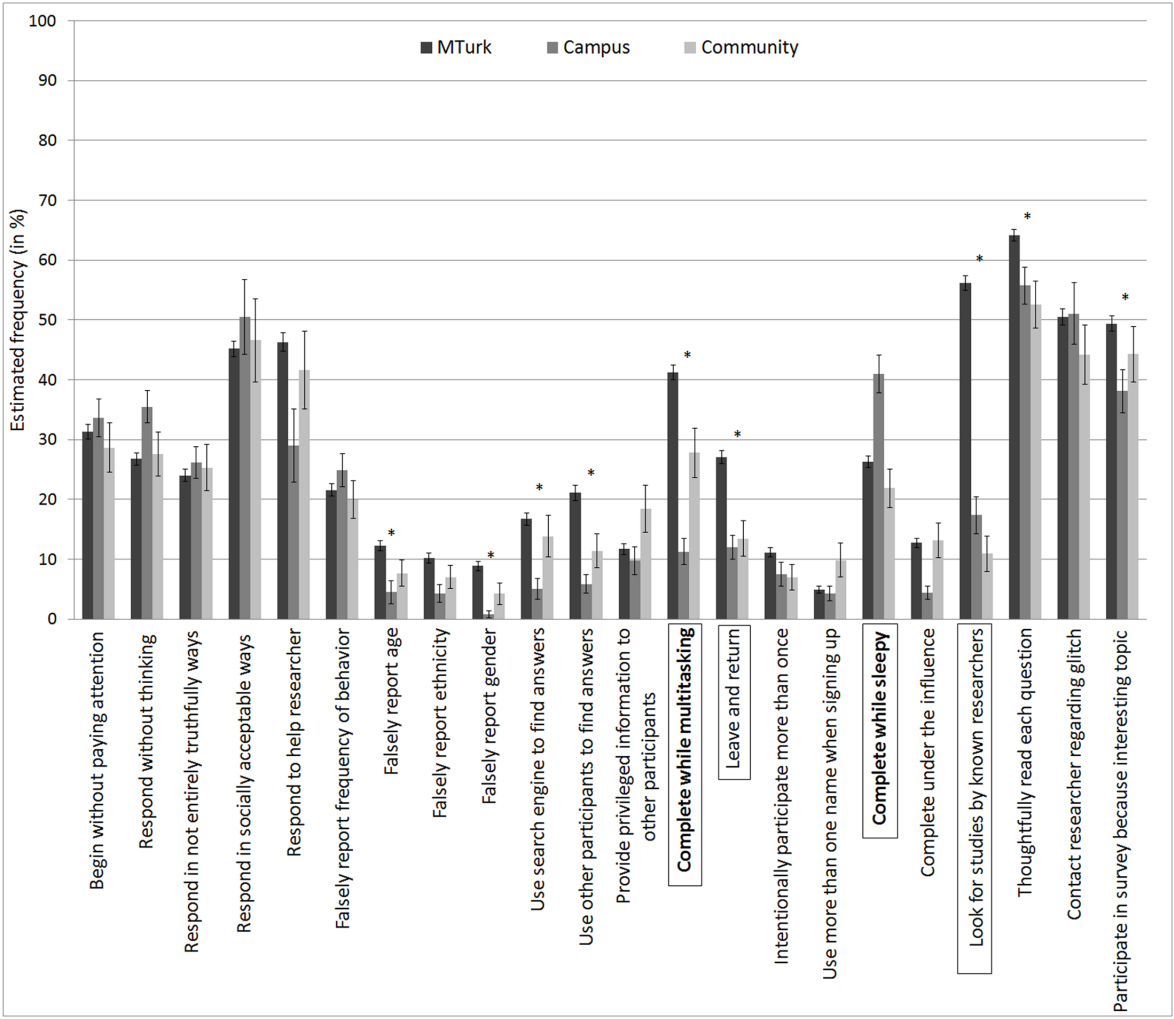
Estimates of the frequency of problematic respondent behaviors based on estimates of others’ behaviors. Error bars represent standard errors. Behaviors for which MTurk participants report greater engagement than more traditional samples are starred. Behaviors for which campus and community samples vary are bolded. Behaviors which vary consistently in both the FO and the FS condition are outlined in a box. Significance was determined after correction for false discovery rate using the Benjamini-Hochberg procedure. Note that frequency estimates are derived in the most conservative manner possible (scoring each range as the lowest point of its range), but analyses are unaffected by this data reduction technique. For complete text of each behavior, see Table 1.

We next compared responses from participants in the FO condition (who provided estimates of others’ behaviors) across samples (Fig 2), under the assumption that the FO condition should be less biased than the FS condition (although note that it is also possible that estimates in the FO condition may reflect estimates of behavior among less qualified participants). In the FO condition, the samples varied significantly on a number of problematic responding behaviors. The first orthogonal contrast, which compared MTurk participants’ responses to responses from participants from more traditional testing environments, revealed that MTurk participants were more likely than campus and community participants to falsely report their age (*t*(510) = -3.52, *p* = 4.76E-4, *d* = .31) and gender (*t*(510) = -3.89, *p* = 1.13E-4, *d* = .34), to use search engines (*t*(510) = -3.57, *p* = 3.96E-4, *d* = .32) or other participants (*t*(510) = -4.51, *p* = 8.19E-6, *d* = .40) to find privileged information about how to complete a task, to complete studies while multitasking (*t*(510) = -7.29, *p* = 1.16E-12, *d* = .65), to leave the page of a study to return at a later point in time (*t*(510) = -5.61, *p* = 3.25E-8, *d* = .50), to look for studies by researchers that they already know (*t*(510) = -14.41, *p* = 9.73E-40, *d* = 1.28), to thoughtfully read each question in a survey (*t*(510) = -4.15, *p* = 3.84E-5, *d* = .37), and to participate in a survey because it is an interesting topic (*t*(510) = -2.98, *p* = .003, *d* = .26). The second orthogonal contrast revealed that campus participants were less likely than community participants to complete studies while multitasking (*t*(510) = -3.20, *p* = .001, *d* = .28), but were more likely than community participants to complete studies while sleepy (*t*(510) = 4.31, *p* = 1.95E-5, *d* = .38).

#### Consistencies Across Conditions

Because we did not undertake statistical comparisons of the two conditions, we are precluded from drawing strong conclusions regarding the extent to which participants responded consistently across conditions. Observation of Figs 1 and 2, however, reveals that MTurk participants, regardless of condition, appear to report more frequently multitasked and left and returned to a study than did participants from more traditional samples, and they were more likely to look for studies by researchers that they knew. While campus participants, regardless of condition, more frequently complete studies while sleepy than do community participants, rates of engagement in potentially problematic respondent behaviors were largely consistent across the two more traditional samples across both conditions. Though our intention in including the FO condition was to obtain less biased estimates of participants’ true rates of engagement in each of the potentially problematic behaviors, all data analyzed here is based upon participant self-report and therefore we cannot verify the objective accuracy of either set of estimates.

### Predictors of potentially problematic respondent behaviors

For each behavior, we hypothesized that respondent’s beliefs about, familiarity with, and reasons for participating in psychological studies might be associated with their tendency to engage in potentially problematic behaviors. To test this, we used these factors as simultaneous predictor terms in a multiple linear regression analysis for each problematic responding behavior. Moreover, we were interested in the extent to which these factors’ predictive strength varied by sample, therefore we used sample as a moderator of each predictor. For each behavior, therefore, the full model included the main effect of sample, the main effects of each predictor, and three two-way interactions between sample and each of the predictors. Because between-sample comparisons of the estimated frequency with which participants engage in problematic behaviors appeared relatively consistent across conditions, we report the FS condition here. However, results are largely consistent in the FO condition (available in the S1 File).

Within the FS condition, participants who reported that they more frequently believed that survey measures assessed meaningful psychological phenomena also reported that they less frequently begin studies without paying attention to instructions (*B* = -3.32, *SE* = .82, *t*(504) = -4.05, *p* = 6.04E-5), complete studies while multitasking (*B* = -4.86, *SE =* 1.08, *t*(504) = -4.49, *p* = 8.79E-6), respond to questions in ways that are not entirely truthful (*B* = -2.22, *SE* = .68, *t*(504) = -3.26, *p* = .001), leave the page of a study and return at a later point in time (*B* = -3.71, *SE* = .69, *t*(504) = -5.39, *p* = 1.07E-7), falsely report their age (*B* = -1.34, *SE* = .47, *t*(504) = -2.87, *p* = .004), and falsely report the frequency with which they engage in certain behaviors (*B* = -1.69, *SE* = .50, *t*(504) = -3.36, *p* = .001). They also reported that they more frequently thoughtfully read each question in a survey (*B* = 3.62, *SE* = .86, *t*(504) = 4.19, *p* = 3.31E-5) and participate in a survey because the topic is interesting (*B* = 5.64, *SE* = 1.33, *t*(504) = 4.23, *p* = 2.80E-5). The association between belief in the meaningfulness of survey measures and engagement in one potentially problematic respondent behavior was actually reversed in community participants such that, relative to MTurk participants, greater belief in the meaningfulness of these measures was associated with more frequent tendency to respond in ways that are not entirely truthful (*B* = 6.94, *SE* = 2.09, *t*(504) = 3.32, *p* = .001).

Participants who reported that they used compensation from MTurk or psychology studies as their primary form of income reported more frequently falsely reporting their age (*B* = 3.95, *SE* = 1.22, *t*(504) = 3.23, *p* = .001), ethnicity (*B* = 3.47, *SE* = 1.09, *t*(504) = 3.20, *p* = .001), and gender (*B* = 2.73, *SE* = .76, *t*(504) = 3.61, *p* = 3.44E-4), providing privileged information on how to complete a task (*B* = 4.78, *SE* = 1.62, *t*(504) = 2.95, *p* = .003), using search engines to find information on how to complete a task (*B* = 5.27, *SE* = 1.61, *t*(504) = 3.27, *p* = .001), using more than one ID when signing up for studies (*B* = 2.90, *SE* = .78, *t*(504) = 3.73, *p* = 2.11E-4), and intentionally participating in the same study more than once (*B* = 3.46, *SE* = 1.17, *t*(504) = 2.94, *p* = .003). Additionally, relative to MTurk participants who use compensation from MTurk as their primary source of income, community participants who use compensation from studies as their primary source of income were more likely to begin studies without paying full attention to instructions (*B* = 25.44, *SE* = 7.77, *t*(504) = 3.28, *p* = .001) and to complete studies under the influence of drugs and alcohol (*B* = 16.43, *SE* = 5.62, *t*(504) = 2.92, *p* = .004). However, only six community members indicated that they used their study compensation as their primary source of income, so results specific to community members are underpowered and should be interpreted cautiously.

Spending more time completing studies or on MTurk was associated with less frequently responding without really thinking about a question (*B* = -2.70, *SE* = .80, *t*(504) = -3.39, *p* = .001), but was not significantly associated with rates of engagement in any other potentially problematic respondent behaviors.

## Discussion

Underpowered research designs can misrepresent true effect sizes, making it difficult to replicate published research even when reported results are true. Recognition of the costs of underpowered research designs has led to the sensible recommendation that scientists make sample size decisions with regard to statistical power (e.g., [38]). In response, many researchers have turned to crowdsourcing sites such as MTurk as an appealing solution to the need for larger samples in behavioral studies. MTurk appears to be a source of high quality and inexpensive data, and effect sizes obtained in the laboratory are comparable to those obtained on MTurk. Yet this is seemingly inconsistent with reports that MTurk participants engage in behaviors which could reasonably be expected to adversely influence effect sizes, such as participant crosstalk (e.g., through forums) and participating in similar studies more than once. One possibility is that laboratory participants are equally likely to engage in behaviors which have troubling implications for the integrity of the data that they provide.

In the present study, we examined the extent to which participants engage in a number of behaviors which could influence data quality and we compared the frequency with which participants engage in such behaviors across samples. The present study suggests that participants tend to engage in behaviors that may be problematic for the integrity of their responses. Importantly, we find relatively few differences in how frequently participants from an MTurk, campus, and community sample engage in these behaviors. As previously demonstrated (e.g., [17]), MTurk participants are somewhat more distracted than participants from non-crowdsourced samples—they are more likely to multitask during studies and to leave the page of a study while they are completing it. Somewhat troublingly, MTurk participants also report that they participate in studies by researchers that they already know more often than do participants from the campus and community. Because researchers tend to conduct multiple studies addressing the same general research question and potentially using the same or similar paradigms, it is imperative that researchers screen for participants who have previously completed studies (as has been highlighted extensively in [13,15], especially because non-naïveté among participants can reduce effect sizes [21]).

Because we were concerned that participants might present an overly rosy image of their behavior, we included a condition in which some participants estimated the frequency with which *other* participants engaged in certain behaviors, reasoning that these estimates would be egocentrically anchored upon their own behaviors but less subject to the influence of self-serving biases. Interestingly, when we asked participants to report on others’ behaviors rather than their own, we observed that MTurk participants reported more frequent engagement in potentially problematic respondent behaviors than traditional participants: they reported more frequently falsifying their gender, age, and ethnicity and seeking out privileged information from search engines or other participants. Although it is possible that, as hypothesized, results from estimates of others’ behaviors reflect a more objective and less biased reality, there are a number of reasons to be cautious about drawing this conclusion. As a function of our eligibility requirements, our MTurk sample was comprised only of highly prolific participants (over 1,000 HITs submitted) who are recognized for providing high-quality data (95% approval rating). Because these eligibility requirements were the default and recommended settings at the time that this study was run [28], we reasoned that most laboratories likely adhered to such requirements and that this would allow us to best sample participants representative of those typically used in academic studies. However, participants were asked to estimate behavioral frequencies for the *average* MTurk participant, who is likely of much poorer quality than were our highly-qualified MTurk participants, and thus their responses may not necessarily reflect unbiased estimates anchored upon their own behavior, calling the accuracy of such estimates into question. Thus, findings which emerged only in reports of others’ behaviors should be considered suggestive but preliminary.

Our results also suggest that a number of factors may influence participants’ tendency to engage in potentially problematic responding behaviors, including their belief that surveys measure meaningful psychological phenomena, their use of compensation from studies as their primary form of income, and the amount of time they typically spend completing studies. Generally, we observed that belief that survey measures assess real phenomena is associated with lower engagement in most problematic respondent behaviors, potentially because participants with this belief also more strongly value their contribution to the scientific process. Community participants who believed that survey measures were assessments of meaningful psychological phenomena, however, were actually *more* likely to engage in the potentially problematic behavior of responding untruthfully. One can speculate as to why community participants exhibit a reversal on this effect: one possibility is that they behave in ways that they believe (falsely) will make their data more useful to researchers without full appreciation of the importance of data integrity, whereas campus participants (perhaps aware of the import of data integrity from their science classes) and MTurk participants (more familiar with the scientific process as a function of their more frequent involvement in studies) do not make this assumption. However, the underlying reasons why community participants exhibit this effect ultimately await empirical investigation.

We also observed that participants who completed more studies generally reported less frequent engagement in potentially problematic respondent behaviors, consistent with what would be predicted by Chandler and colleagues’ (2014) [15] findings that more prolific participants are less distracted and more involved with research than less prolific participants. Our results suggest that participants who use compensation from studies or MTurk as their primary form of income report more frequent engagement in problematic respondent behaviors, potentially reflecting a qualitative difference in motivations and behavior between participants who depend on studies to cover their basic costs of living and those who do not. Importantly, while using compensation from studies as one’s primary form of income and spending more time completing studies were associated with differential rates of engagement in potentially problematic respondent behaviors, these factors had predictive power for far fewer of the potentially problematic respondent behaviors than beliefs about survey measures did.

It is worth considering if there might be additional reasons why participants engage in problematic respondent behaviors. Though statistical analyses were not conducted on participants’ free-response data, inspection of these responses suggested that participants may not believe that their problematic behaviors are all that problematic and may even be beneficial (for instance, they may listen to music while completing studies, which we have considered a form of potentially detrimental multitasking, for the express purposes of improving their concentration). Participants also reported that they primarily comply with researcher requests to minimize interruptions and distractions when such requests are made, but that such requests are rare. Because answering questions can be boring and participants are paid by how many studies they complete, participants may respond to incentives to complete studies hurriedly and inattentively, and engaging in dishonest behavior to access some (e.g., well-paying) studies or simply to break the tedium of completing studies.

It is important to note also that these analyses are correlational. Thus, an interpretation that those participants with certain beliefs about the meaningfulness of survey measures will behave in a certain way, for example, or an alternative interpretation that participants who behave in a certain way will develop beliefs about survey measures, are equally likely. Our intention in including such analyses was to help researchers understand the characteristics of individuals who engage in greater rates of potentially problematic respondent behaviors, so that they might assess the extent to which these factors are associated with their own effects. For example, if one observes a strong association variables *x* and *y*, but variable *x* is also strongly associated with participants’ beliefs about the meaningfulness of survey measures, one might consider whether the same pattern of responses in variable *y* could be explained by participant engagement in potentially problematic respondent behaviors that are more frequent among those who believe survey measures are valid assessments of psychological phenomena.

Because variables such as subject pool, sampling procedures, time of day, and experimental controls all contribute to heterogeneity in observed effect sizes [39], participants’ problematic behavior while completing studies has strong potential to influence data accuracy. One way in which it may do so is by simply increasing the random error of a sample. Inattentive responding, participating under the influence, and falsifying responses to survey measures may simply increase the variance of a given estimate. However, through the law of large numbers, the influence of such noise should decrease with increasing sample size. Alternatively, some behaviors may systematically bias the data which participants provide. Lying about demographic variables, for instance, may bias effect sizes in designs that use demographic variables are quasi-independent factors. As an example, a researcher studying implicit gender attitudes might observe somewhat muted effects if some portion of the sample falsely reported their gender. Additionally, behaviors such as participants’ exchange of information with other participants, online search for information about tasks, and previous completion of tasks all influence the level of knowledge of the experimental task that any given participant has, leading to a non-naïveté that can bias results [21,40]. Unlike random noise, the impact of systematic bias increases as sample size increases. It is therefore this latter set of behaviors that have the potential to be particularly pernicious in our attempts to measure true effect sizes and should most ardently be addressed with future methodological developments.

However, the extent to which these behaviors are ultimately problematic with regards to their impact on data quality is still uncertain, and is certainly a topic worth future investigation. Our intention here was to highlight the range of behaviors that participants in various samples might engage in, and the relative frequency with which they occur, so that researchers can make more informed decisions about which testing environment or sample is best for their study. If a researcher at all suspects that these potentially problematic behaviors might systematically influence their results, they might want to avoid data collection in those populations. As one example, because MTurk participants multitask while completing studies with relatively greater frequency than other populations, odds are higher among an MTurk sample that at least some participants are listening to music, which may be problematic for a researcher attempting to induce a mood manipulation, for example. Although a great deal of recent attention has focused on preventing researchers from using questionable research practices which may influence estimates of effect size, such as making arbitrary sample size decisions and concealing non-significant data or conditions (c.f., [22,38]), every decision that a researcher makes while designing and conducting a study, even those that are not overtly questionable such as sample selection, can influence the effect size that is obtained from the study. The present findings may help researchers make decisions regarding subject pool and sampling procedures which minimize the likelihood that participants engage in problematic respondent behaviors which have the potential to impact the robustness of the data that they provide.

Yet the present findings are subject to a number of limitations. In particular, a number of our items were worded such that participants may have interpreted them differently than we intended, and thus their responses may not reflect engagement in problematic behaviors, per se. For instance, participants may indeed *not* ‘thoughtfully read each item in a survey before answering’, simply because most surveys include some demographic items (e.g., age, sex) which do not require thoughtful consideration. Participants may not understand what a hypothesis is, or how their behavior can impact a researchers’ ability to find support for their hypothesis, and thus responses to this item may be subject to error. The scale with which we asked participants to respond may also have introduced confusion, particularly to the extent to which participants had trouble estimating how frequently they engage in a certain behavior out of all of the *time* they spend on MTurk or completing studies (rather than, for instance, how frequently they’ve engaged in a behavior out of all of the *number* of studies they have completed) and then converting that frequency to a percentage. These concerns with our measurement instrument call into question the accuracy of the absolute frequencies with which participants report engaging in some behaviors. Thus, while researchers can use absolute frequency estimates in order to approximate generally whether engagement in these behaviors is low or high, limitations inherent in our measurement instrument may make consideration of the *relative* rates of engagement in these behaviors between samples more appropriate when making decisions regarding sample population. Additionally, because we only had adequate statistical power, (1- *β*) = .80, to detect medium-sized between-samples effects, small effects should be taken as provisional and awaiting replication.

By administering the present study to campus and community participants in a physical lab environment, we’ve confounded mode of survey administration and sample in our between-sample comparisons. Researchers often compare laboratory-based samples (comprised of participants who complete studies in a physical lab environment) to crowdsourced samples (comprised of participants who, by necessity, complete studies in an online environment) and obtain comparable effects (e.g., [11]). Thus, we were interested in comparing how frequently MTurk, campus, and community participants reported engaging in potentially problematic respondent behaviors while completing a *typical* study (e.g., an online study for MTurk participants and a study in a physical lab environment for campus and community samples), as we expected that this comparison would be most informative to researchers making decisions regarding which sample to utilize. However, engagement in potentially problematic respondent behaviors varies among campus-based populations as a function of whether they complete studies in a physical testing environment or online [41], and thus the extent to which MTurk participants’ greater engagement in some problematic respondent behaviors is a characteristic of crowdsourced samples or is simply a function of them completing studies online is presently unknown. Our results may therefore be less informative to a researcher trying, for example, to decide between MTurk and an online survey using campus participants. Yet these limitations primarily pertain to interpretation of significant comparisons between samples, of which there were few. That significant differences of at least medium effect size between samples were relatively few is compelling, suggesting that the potential operation of experimental artifacts is not unique to crowdsourcing sites.

In sum, though many of these potentially problematic behaviors are familiar to researchers and methods have been developed to address these confounding influences, these methods may not be entirely suitable for addressing all of the problematic respondent behaviors in which participants can engage or may not be readily applied by researchers. Online research using crowdsourcing sites presents new challenges for achieving experimental control, and yet we must not forget the importance of such controls in more traditional campus- and community-based settings. The present study suggests that participants engage in potentially problematic respondent behaviors at non-zero frequencies, and that rates of engagement in many of these behaviors do not vary by sample. It is thus important to consider how participants’ potential engagement in these problematic respondent behaviors might influence the integrity of data that they provide. Making sample sizes decisions based on statistical power may increase the likelihood of detecting true effect sizes, but only when due attention is given to the operation of experimental artifacts and problematic respondent behaviors.

## Supporting Information

S1 File Supplementary Materials. Supplementary materials include predictors of problematic respondent behaviors in the FO Condition and qualitative summaries of participants’ explanations for engagement in potentially problematic respondent behaviors.
(PDF)
