# Supplementary material for: Measuring the Prevalence of Problematic Respondent Behaviors among MTurk, Campus, and Community Participants
Source: PLoS One. 2016 Jun 28;11(6):e0157732. doi: 10.1371/journal.pone.0157732 (PMC4924794; doi:10.1371/journal.pone.0157732)
Supplement: S1 File — Supplementary materials include predictors of problematic respondent behaviors in the FO Condition and qualitative summaries of participants’ explanations for engagement in potentially problematic respondent behaviors. (PDF) [file pone.0157732.s001.pdf]

Supplementary Materials:

Measuring the prevalence of problematic respondent behaviors among MTurk, campus, and community participants

Elizabeth A. Necka, Stephanie Cacioppo, Greg J. Norman, and John T. Cacioppo

Predictors of potentially problematic respondent behaviors

FO Condition. If we assume that estimates of the problematic behaviors identified in the FO condition are anchored on a respondent's own behavior, then we also expect that respondent's estimates of others' beliefs about, familiarity with, and reasons for participating in psychological studies should be anchored on their own beliefs, familiarity, and reasons (although as reported in the main text, there may be alternative mechanisms driving participants' responses in the FO condition). We hypothesized that these factors might be related to problematic responding behaviors; therefore, we used these as simultaneous predictor terms in a multiple linear regression analysis for each problematic responding behavior. Moreover, we were interested in the extent to which these factors' predictive strength varied by sample, therefore we used sample as a moderator of each predictor. As reported in the main text, we also included a covariate which distinguished between participants who answered both numerical ability questions correctly and those who did not, thus controlling for participants' numerical abilities. For each behavior, therefore, the full model included the main effect of sample, the main effects of each predictor, the covariate of numerical ability, and three two-way interactions between sample and each of the predictors.

Analyses revealed that the more meaningful psychological research is believed to be, the less frequent are the problematic responding behaviors of beginning studies without paying attention to instructions (B = -4.53, SE = 1.18, $t(501)$ = -3.85, p = 1.31E-4), completing studies while multitasking (B = -5.13, SE = 1.21, $t(501)$ = -4.23, p = 2.77E-5), responding to questions in ways that are not entirely truthful (B = -3.13, SE = 1.05, $t(501)$ = -2.98, p = .003), responding without really thinking about a question (B = -5.19, SE = 1.04, $t(501)$ = -4.99, p = 8.04E-7), falsely reporting one's age (B = -3.31, SE = .79, $t(501)$ = -4.18, p = 3.49E-5) and ethnicity (B = -2.37, SE = .79, $t(501)$ = -3.02, p = .003), falsely reporting the frequency with which they engage in certain behaviors (B = -3.70, SE = 1.01, $t(501)$ = -3.67, p = 2.38E-4), intentionally participating in the same study more than once (B= -2.37, SE = .78, $t(501)$ = -3.03, p = .003), using a search engine to find the answer to a survey or the key to an experimental task (B = -2.77, SE = .98, $t(501)$ = -2.82, p = .005), and completing studies under the influence of alcohol or

drugs (*B* = -3.20, *SE* = .79, *t*(501) = -4.03, *p* = 6.46E-5), and the more frequent are the behaviors of thoughtfully reading each question in a survey (*B* = 7.01, *SE* = .93, *t*(501) = 7.50, *p* = 2.88E-13) , responding in ways that help the researcher find support for his or her hypotheses (*B* = 4.70, *SE* = 1.50, *t*(456) = 3.13, *p* = .002), and participating in a survey because the topic is interesting (*B* = 6.03, *SE* = 1.24, *t*(501) = 4.88, *p* = 1.42E-6). A significant interaction emerged for one practice, such that community participants, relative to the MTurk sample, who reported greater belief that surveys assess meaningful psychological phenomena also reported that the average participant engaged in even lower rates of using more than one ID when completing studies (e.g., the negative correlation was stronger; *B* = -4.80, *SE* = 1.60, *t*(501) = -2.99, *p* = .003).

Reporting that a greater percentage of participants used compensation from MTurk/studies as their primary form of income was associated with reporting that the average participant more frequently providing privileged information to other participants (*B* = 3.23, *SE* = 1.07, *t*(501) = 3.01, *p* = .003), using more than one ID when completing studies (*B* = 2.18, *SE* = .68, *t*(501) = 3.18, *p* = .002), and intentionally participating in the same study more than once (*B* = -2.80, *SE* = .92, *t*(501) = -3.05, *p* = .002). Significant interactions emerged for two practices: relative to the MTurk sample, community participants reporting that a greater percentage of participants use compensation from studies as their primary source of income also reported that the average participant engaged in lower rates of the problematic behaviors providing privileged information to other participants (*B* = -6.61, *SE* = 2.31, *t*(501) = -2.86, *p* = .004) and using more than one ID to participate in studies (*B* = -4.47, *SE* = 1.47, *t*(501) = -3.04, *p* = .003).

Reporting that the average participant spends more time completing studies was associated with reporting that the average participant less frequently intentionally participates in the same study more than once (*B* = -2.80, *SE* = .92, *t*(501) = -3.36, *p* = .001) and more frequently contacts the researcher if there is a glitch in their survey (*B* = 4.96, *SE* = 1.52, *t*(501) = 3.25, *p* = .001) and looks for studies by researchers that they already know (*B* = 4.54, *SE* = 1.30, *t*(501) = 3.48, *p* = .001).

Participants' explanations for engagement in potentially problematic respondent behaviors

We provided participants with the opportunity to explain in a free-response text box why or why not engaging in each of the potentially problematic respondent behaviors would be considered defensible behavior. Because participants were not required to provide these responses, the responses which were collected suffer from a selection bias and thus were not included in the main text. Further, very few campus and community participants provided responses to these items. However, examination of such responses may provide a more nuanced understanding of the motivations underlying participants' behavior in potentially problematic respondent behaviors. This may be particularly informative for understanding MTurk participants' behaviors, which are not directly examinable in the testing environment. Thus, we provide qualitative inspection of these responses here. For each behavior, the first author (E.A.N.) read each response, created categories which appeared to fit the qualitative responses, and then categorized each response into one or more categories. Note that inspection and categorization of responses was conducted after statistical analysis of the frequency estimates by sample, and thus the author was not blind to results. Rank order of the categories for each behavior are described here, though counts of the number of responses in each category are omitted, as many responses touched on more than one qualitatively distinct topic and thus were counted in more than one category. Here, we present categorical descriptions of the responses from MTurk participants for each behavior which varied significantly by sample in either the FS or FO condition. We collapse responses across condition (FS or FO) for the purpose of qualitative summary. For the interested reader, the full dataset (including all raw qualitative responses) can be found at https://dataverse.harvard.edu/dataverse/MeasuringPPRBs.

Completing studies while multitasking. MTurk participants report multitasking primarily by listening to music, and that they report that they can still concentrate while they listen to music. This was the most frequent free-response. The second most frequent response was a report that some types of multitasking actually help participants focus, and the third most frequent response was that multitasking is necessary in order to complete surveys which have boring, repetitive, or not intellectually demanding questions.

Leaving the page of a study and returning. MTurk participants' most frequent response was that they leave and returning to a page in a survey primarily when they have an unexpected interruption (e.g., an emergency, needing to use the restroom, a phone call). The second most frequent reason why participants reported that it would be defensible to leave and return to a page of a survey is that researchers do not specify that participants should complete the study in one sitting, and the third most common reason is that participants get bored or distracted while completing long surveys.

Completing studies while sleepy. MTurk participants primarily report that being sleepy while completing studies is acceptable as long as participants are alert 'enough', which they report is a judgment call that the participant has to make based on their own sleepiness and the requirements of their task. The second most common response was that responding to surveys while sleepy was never okay, either because it leads to inaccurate data or because it wastes participants' time. The third most common response regarding the defensibility of completing studies while sleepy is that most people are sleepy at some point during their work and it is unrealistic to expect that MTurk participants will be performing at their full capacity all of the time.

Looking for a study by a researcher one already knows. Overwhelmingly, MTurk participants report primarily looking for studies by a known researcher because they know that the researcher will be fair and timely in compensating them (the count for this response was nearly eightfold greater than the count for the second most frequent response). The second most frequent reason that participants identified as a defensible reason for looking for studies by a known researcher is that they knew that they would know how to complete the studies (because they had completed similar ones in the past) and that they liked the researcher or enjoyed the topic or type of study that the researcher primarily posts.

Contacting a researcher if there is a glitch in their survey. MTurk participants primarily report contacting the researcher regarding a glitch in the survey as a way to help the researcher. The second most reported reason was to ensure that they received adequate payment for their time and the third most common reason was to ensure that the site remained in good working condition for all other MTurk participants.

Falsely reporting one's age. MTurk participants primarily responded by indicating that falsely reporting one's age is unacceptable because it will adversely affect the data and the researcher's conclusions. The second most common response was that participants will do what is necessary in order to access surveys (e.g., lie about one's age in order to qualify), and the third most common response was that reporting that one's age is different than what it is by only a 'few' years is acceptable because it is unlikely to adversely affect data.

Falsely reporting one's gender. MTurk participants primarily reported that under no circumstances was there an acceptable reason for one to falsely report one's gender. They also reported second most frequently that falsely reporting one's gender can have adverse implications for the researcher's findings and thus should be avoided. The third most frequent response was that participants falsely report their gender in order to qualify for certain studies which indicate gender as qualification criterion.

Using other participants to find answers to questions or how to complete a survey. The overwhelming response from MTurk participants (over five-fold more frequent than the next most frequent response) was that it is acceptable only to use forums or other participants to find information about how to complete a survey (e.g., in the case of ambiguous instructions or a technical error) or about how long a survey takes or how long it pays, rather than to find out information about how to answer a question. The second most common response was that speaking with other participants is considered cheating and is unethical and unacceptable, and the third most common response was that speaking to other participants should be avoided because it can adversely affect data.

Thoughtfully reading each question in a survey. Participants were most likely to indicate that they should thoughtfully read each question in a survey because they are being paid to do this or that MTurk was their job and that this is an expectation of the job responsibilities. The second most frequent response was that it is necessary to thoughtfully read each question in a survey in order to avoid rejection and ensure accurate responses to all attention check items. The third most common response was that they

thoughtfully read each question in a survey because they understand that this is necessary in order to provide the researchers with accurate data.

Participating in a survey because it is an interesting topic. MTurk participants reported first most commonly that they participate in studies that are of interest to them because doing so increases their motivation and attention to the survey. Their second most common response for why this behavior is defensible was that this makes it easier to get through more boring surveys (e.g., by placing an interesting survey amidst many less interesting ones). Finally, they also reported that they enjoy learning new information from their time taking surveys.
